# Evaluation of the Antibacterial Activity of Eco-Friendly Hybrid Composites on the Base of Oyster Shell Powder Modified by Metal Ions and LLDPE

**DOI:** 10.3390/polym14153001

**Published:** 2022-07-25

**Authors:** Januar Widakdo, Tsan-Ming Chen, Meng-Chieh Lin, Jia-Hao Wu, Tse-Ling Lin, Pin-Ju Yu, Wei-Song Hung, Kueir-Rarn Lee

**Affiliations:** 1 Advanced Membrane Materials Research Center, Graduate Institute of Applied Science and Technology, National Taiwan University of Science and Technology, Taipei 106335, Taiwan; januarwidakdo@gmail.com; 2 Carbide Division, Formosa Plastics Corporation, Taipei 105076, Taiwan; tm-chen@fpc.com.tw (T.-M.C.); mjlin373@fpc.com.tw (M.-C.L.); g9651359@gmail.com (J.-H.W.); rachellin@fpc.com.tw (T.-L.L.); ruru0709@fpc.com.tw (P.-J.Y.); 3 R&D Center for Membrane Technology, Department of Chemical Engineering, Chung Yuan University, Chungli 32023, Taiwan

**Keywords:** antibacterial material, biomaterials, calcium oxide, *S. aureus*, *E. coli*

## Abstract

Transforming biological waste into high-value-added materials is currently attracting extensive research interest in the medical and industrial treatment fields. The design and use of new antibacterial systems are urgently needed. In this study, we used discarded oyster shell powder (OSP) to prepare calcium oxide (CaO). CaO was mixed with silver (Ag), zinc (Zn), and copper (Cu) ions as a controlled release and antibacterial system to test the antibacterial activity. The inhibition zones of various modified metals were between 22 and 29 mm for *Escherichia coli* (*E. coli*) and between 21 and 24 mm for *Staphylococcus aureus* (*S. aureus*). In addition, linear low-density polyethylene (LLDPE) combined with CaO and metal ion forms can be an excellent alternative to a hybrid composite. The strength modulus at 1% LLDPE to LLDPE/CaO Ag increased from 297 to 320 MPa. In addition, the antimicrobial activity of LLDPE/CaO/metal ions against E. coli had an antibacterial effect of about 99.9%. Therefore, this hybrid composite material has good potential as an antibacterial therapy and biomaterial suitable for many applications.

## 1. Introduction

Microbial infection on polymer-based biomedical appliances appears to be a significant threat within the healthcare industry as it can cause infectious outbreaks and economic losses. Gram-positive *Staphylococcus aureus* (*S. aureus*) and gram-negative *Escherichia coli* (*E. coli*) are the two major nosocomial pathogens that can lead to a broad spectrum of infections, including skin and soft tissue infections, surgical site infections (SSI), catheter-related infections, septic shock, pneumonia, endocarditis, bacteremia, and cellulitis. Nevertheless, bacterial resistance to antibiotics and their dissemination has resulted in significant health problems, leading to treatment drawbacks for many drugs.

At present, antibacterial materials can be roughly divided into two types: inorganic antibacterial agents, such as aluminum (Al) [1,2,3,4], silver (Ag) [5,6,7,8,9], copper (Cu) [10,11,12], magnesium (Mg) [13], and zinc (Zn) [13,14,15,16,17,18]), and organic antibacterial agents (chlorhexidine diacetate [19,20], triclosan [21], polyaniline [22], and polyethyleneimine [23,24]). Among the two types, inorganic antibacterial agents have better characteristics due to their excellent heat resistance, high hardness, and excellent chemical resistance [25,26]. Therefore, in practical applications, inorganic antibacterial agents are currently the mainstream. Inorganic materials mainly achieve antibacterial effects through physical adsorption or ion exchange, and are usually metal nanoparticles and their compounds. Among many metal nanomaterials, mercury, silver, cadmium, copper, zinc, etc., have a strong antibacterial ability. Still, the safe and nontoxic ones are limited to silver, zinc, and copper ions, and the antibacterial ability of silver ions is much stronger than that of zinc, copper, and other metal ions. Therefore, inorganic silver-based antibacterial agents occupy the dominant position of inorganic antibacterial agents.

The conversion of biological waste into high-value-added materials has attracted extensive research interest [27]. Since Taiwan is surrounded by sea aquaculture, the pollution and biological waste caused by aquaculture are considerable. According to statistics from the Taiwan Agricultural Commission (ROC), the average annual production of oyster shells (OS) in Taiwan has exceeded 160,000 metric tons over the past decade. These wastes are mainly treated as roadbed or embankment materials, feed additives for poultry, and soil additives. Since these applications are inefficient, greater emphasis must be placed on exploring more efficient waste recycling methods [28]. Utilizing fishery by-products can increase fishery products’ total economic value while reducing waste. Some examples include the production of chitosan from shrimp or crab shells, heavy metal absorption or biomass fermentation using algal residues [29,30], collagen extraction from fish scales, and the fermentation of squid guts to make soy sauce [31]. The shells of shellfish, such as oyster shells are rich in calcium carbonate (CaCO_3_), which can be converted into calcium oxide (CaO) in the shell waste through heat treatment (calcination or pyrolysis). CaO triggers excellent antibacterial activity [32].

The importance of CaO nanoparticles is stressed, as they are biocompatible and very promising antibacterial agents [33]. CaO nanoparticles can eliminate 99% of pathogens such as *E. coli* when they are exposed to 0.05% of these particles [34]. They presented bactericidal action against *Salmonella typhimurium*, *Staphylococcus aureus*, and *Bacillus subtilis* [34,35,36]. Another advantage of the biocidal behavior of CaO is that the mechanism is not only related to the generation of reactive oxygen species (ROS) on the surface, as for other metal oxides, but also to the pH increase by hydration, forming hydroxides and releasing Ca^2+^ ions [37,38]. Gedanken et al. [39] also studied the bactericidal effect of CaO nanoparticles on gram-positive (*S. aureus*) and gram-negative (*E. coli*) bacteria, showing these possible mechanisms. However, there are still many inconveniences in the practical application of CaO oyster shell powder (OSP). Recent studies have combined polymers with oyster shell powder to give it a more expansive application space. Tsou et al. [40] incorporated OSP calcined at 800 °C into processed, modified polyethylene (MPP) to make a composite material with antibacterial function, which could inhibit *S. aureus* [40].

Polyolefins like polyethylene (PE) and polypropylene (PP) have been utilized extensively in business and industry. One of them, linear low-density polyethylene (LLDPE), has decent impact resistance and tensile strength [41]. LLDPE has low crystallinity, which contributes to its excellent transparency [42]. LLDPE’s applicability is nonetheless constrained because it is a nonpolar polymer with an inert surface and low surface energy. Therefore, when creating LLDPE composite materials, we need to add fillers to adjust their properties. Due to CaO being used as bactericides, adsorbents, and, in particular, as destructive adsorbents for a toxic chemical agent, it has become a promising candidate for developing biocomposites [43]. The combination of CaO and LLDPE can improve the antibacterial property, strength, and polarity of LLDPE to a certain extent and help prolong the products’ service life. Therefore, it is crucial to determine how to uniformly disperse CaO into the nonpolar polyolefin matrix and form a good interface to improve the reinforcement effect of LLDPE. Moreover, adding different metal ions can enhance the antibacterial properties of *E. coli* (gram-negative) and *S. Aureus* (gram-positive).

This study aims to combine oyster shell CaO with different metal ions (Ag, Zn, and Cu), then mix it with LLDPE to improve the structural properties of the material and increase the surface mobility and antibacterial properties. This research is expected to discuss the physical and chemical properties of LLDPE added to oyster shell powder, which has been modified using metal ions, as well as optimization of the parameter adjustments of antibacterial ability.

## 2. Materials and Methods

### 2.1. Materials

The following materials were purchased: calcium oxide (CaO, 99.9%) (Formosa, Taipei City, Taiwan), calcium carbonate (CaCO_3_, 99.0%) (Formosa, Taipei City, Taiwan), silver nitrate (AgNO_3_, 99%) (Honeywell Fluka, Seelze, Germany), zinc nitrate hexahydrate (Zn(NO_3_)_2_·6H_2_O, 99%) (Alfa Aesar, Tewksbury, MA, USA), copper (II) chloride, anhydrous (CuCl, >98%) (Alfa Aesar, Tewksbury, MA, USA), sodium carbonate solution (Na_2_CO_3_, 90–100%) (Honeywell Fluka, Seelze, Germany), ammonia solution (NH_3_, 25%) (Honeywell Fluka, Seelze, Germany). All solutions were prepared using deionized water (DI H_2_O, ≥18.2 mΩ-cm). Formosa Plastics Corporation Taiwan provided TAISOX^®^ 3470 Linear low-density polyethylene (LLDPE) used in this research.

### 2.2. Methods

#### 2.2.1. Preparation Process of Metal Ion (Me)-Containing Oyster Shell Powder (OSP)

**Figure 1:**
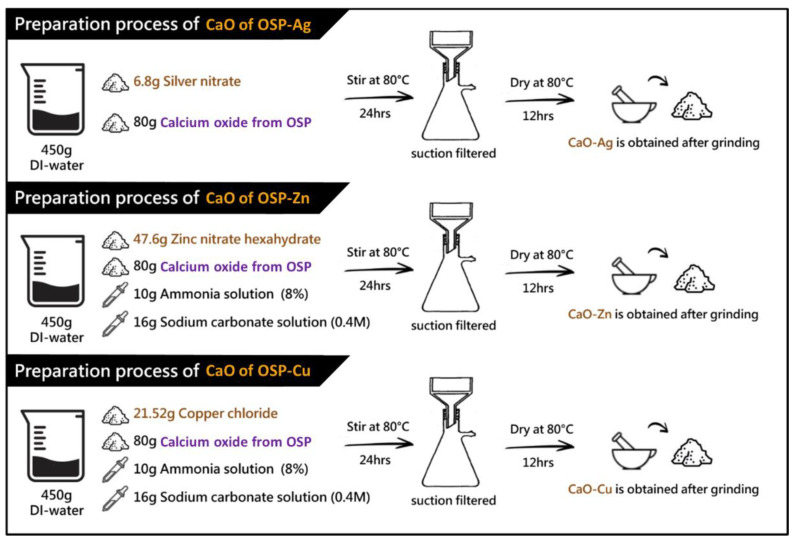
Schematic diagram of the preparation process of metal ion (Me)-containing calcium oxide from oyster shell powder (OSP).

In this study, CaO was produced from oyster shell powder (OSP). Calcinate OSP at a temperature of 800 °C produces a powder product rich in CaO concentration. The process of forming CaCO_3_ from OSP to CaO is shown in Figure S1. After that, the preparation steps for the process of oyster shell powder of CaO containing different metal ions are shown in Figure 1. The step preparation of CaO from OSP mixed with metal ions (Me) is also explained in Section 2.2.1.

##### Preparation Steps of Silver-Containing OSP

Mix 6.8 g of silver nitrate and 80 g of calcium oxide from OSP in 450 g of distilled water, drop in 10 g of ammonia (8%), and 16 g of sodium carbonate solution (0.4 M), and stir at 80 °C for 24 h. Suction filtration was performed for 3 h, and the precipitate was washed and dried in an 80 °C oven for 12 h. The dried powder is ground into fine powder to obtain the finished silver-containing calcium oxide [39,44].

##### Preparation Steps of Zinc-Containing OSP

Mix 47.6 g of zinc nitrate and 80 g of calcium oxide from OSP in 450 g of distilled water, drop in 10 g of ammonia (8%), and 16 g of sodium carbonate solution (0.4 M) at 80 °C for 24 h. Suction filtration was performed for 3 h, and the precipitate was washed and dried in an 80 °C oven for 12 h. The dried powder is ground into fine powder to obtain the finished product containing zinc oxide [45].

##### Preparation Steps of Copper-Containing OSP

Mix 21.52 g of copper chloride, 80 g of calcium oxide from OSP, and 8 g of ascorbic acid in 450 g of distilled water. Add 10 g of ammonia (8%) and 16 g of sodium carbonate solution (0.4 M) dropwise and stir at 80 °C for 24 h. Suction filtration was performed for 3 h, and the precipitate was washed and dried in an 80 °C oven for 12 h. The dried powder is ground into fine powder to obtain the finished copper-containing calcium oxide [46].

#### 2.2.2. The Manufacture of a Sheet of LLDPE Mixed with CaO from OSP and Metal Ions (Me)

LLDPE-CaO metal ion nanocomposites were prepared by melt compounding. The prepared formulations of different compositions are listed in Table S1. The manufacture of a sheet of LLDPE mixed with CaO and metal ions is carried out in 3 stages: the first is mixing LLDPE with a calculated amount of CaO, CaO-Ag, CaO-Zn, and CaO-Cu using a Hydraulic Ram Style Dispersion Kneader Mixer Machine (KD-3-20) with processing temperature at 150 °C, Batch size 3 kg, and Batch mixing time at 10 min. The second stage uses the used BLUEROCK Model (WS-260) Motorized Copper Wire Stripping Machine for crushing the rubber block after processing by the mixer. The last step is to use the plastic injection molding machine SC-110 to produce injection molding test pieces of shell powder composite master batch mixture. Processing temperature at 200 °C and injection pressure (primary pressure/secondary pressure) at 300/200 Kgf/cm^2^. All machines used were provided by Formosa Plastics Corporation Taiwan.

#### 2.2.3. Characterization

##### Wide-Angle X-ray Diffraction

Wide-angle X-ray diffraction (WAXRD) patterns were obtained using a Rigaku diffractometer (model RU-H3R). An X-ray beam based on Ni-filtered Cu-Kα radiation from a sealed tube was operated at 60 kV and 300 mA. Data were collected in the 2θ range of 10–70° with a scanning interval of 0.02.

##### Thermogravimetric Analysis

Thermogravimetric analysis (TGA) was performed using a PerkinElmer TGA (model Pyris 1). The nanocomposite samples (8–10 mg) were heated from 105 °C to 600 °C under nitrogen at a rate of 10 °C/min.

##### Morphological Analysis

A scanning electron microscope (SEM-EDX, JSM-6900LV, JEOL, Tokyo, Japan) was used to analyze LLDPE-OSP-Me hybrid composite morphology with 15 mm × 15 mm × 1 mm, which were placed on a holder fastened with a conductive adhesive tape. Before SEM examination, samples were coated with a thin layer of gold at 15 kV for 20 s to increase the image resolution. Micrographs were taken at 1 K magnifications.

##### The Dynamic Mechanical Analysis (DMA)

The dynamic mechanical analysis (DMA) was carried out using a TA Instruments Q800 DMA. All the samples were measured in a tensile mode over the temperature range of −40 to 150 °C at a heating rate of 3 °C/min and upon a frequency of 1 Hz. Specimen dimensions were 60 × 4 × 0.3 mm^3^.

##### Differential Scanning Calorimetry (DSC)

Differential scanning calorimetry (DSC) was performed using a DSC (model Jade, PerkinElmer, Buckinghamshire, UK). The nanocomposites samples were sealed in an aluminum pan. Scans (55–185 °C) were performed at a heating rate of 10 °C/min under nitrogen purging. The maximum peak in the second scan of the endothermic transition was recorded as the melting point; samples of 7–8 mg in size were used for all of the scans.

#### 2.2.4. Antibacterial Evaluation

##### Microorganisms

*E. coli* 745 and *S. aureus* 9779 were obtained from the Formosa plastics corporation, Taiwan. The strains of bacteria were stored in 20% glycerol solution at −85 °C. They were thawed and incubated in brain heart infusion broth (Eiken Chemicals, Tokyo, Japan) at 37 °C for 20 h. The cells were in the stationary phase and washed once in sterile saline (0.85 *w*/*v*%), and then resuspended in saline at approximately 10^5^ CFU/mL. The tube containing the bacterial suspension was immersed in ice water before use in the experiments.

##### Antibacterial Evaluation

The antibacterial activity of the LLDPE-CaO-Me hybrid composite was tested against *Escherichia coli* (*E. coli*) and *Staphylococcus aureus* (*S. aureus*) (quantitative evaluations). In the quantitative test (ASTM E2149-01), each LLDPE-CaO-Me hybrid composite chip (2 g) was inoculated with a suspension in a serum bottle containing 50 mL of a bacterial liquid with a concentration of about 1 × 10^5^ CFU/mL. It was shaken at 37 °C for 18 h, and then the number of bacteria was counted.

## 3. Results and Discussion

### 3.1. Metal-Containing Calcium Oxide Powder

**Figure 2:**
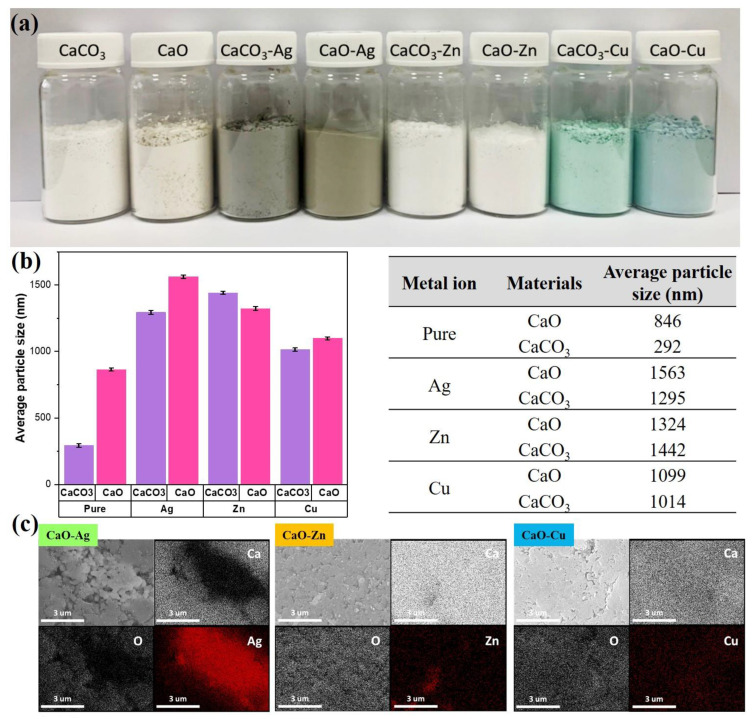
(**a**) Calcium carbonate and calcium oxide from OSP-modified metal ion powder image, (**b**) average particle size analysis of a series of modified metal ion powders of calcium carbonate and calcium oxide. (**c**) EDX spectra of CaO-containing metal ions (Ag, Zn, Cu).

The resulting photos of calcium carbonate (CaCO_3_) and calcium oxide (CaO) from OSP, which have been modified with three metal ions (Ag, Zn, and Cu), are shown in Figure 2a. From the appearance of the powder before and after modification, the difference is quite significant. The pure CaCO_3_ and CaO, before being modified, look like a cream-colored powder. After being modified using Ag metal ions, the color of the powder changed to a grayish brown. Likewise, after CaCO_3_ and CaO were modified using Cu metal ions, the powder color changed to a bluish-green. Furthermore, the color of the powder also changed when added with Zn metal ions. The powder looked white.

The next step was to analyze the average particle size of the modified metal ion powder through a particle size analyzer. The results are shown in Figure 2b. First, comparing CaCO_3_ and sintering results showed that the average particle size of CaCO_3_ was 292 nm, and the average particle size of CaO after sintering was 864 nm. The larger mean particle size of CaO can be attributed to the high-temperature sintering process caused by particle aggregation. Further observation of the powders modified by metal ions found that the average particle size of powders increased, and the particle size distribution was between 1014 and 1563 nm, resulting in an increase in the average particle size of powders after metal modification. It is estimated that there are two points. The first point is that the modified metal particles may be coated on a porous carrier, increasing the average particle size after the overall modification. Second, it is estimated that there are various metal ions in the liquid agglomeration. Thus, the accumulation of the CaCO_3_ and CaO powders during filtration and drying after phase modification increases the average particle size.

After analyzing the physical average particle size, the next step was to analyze the chemical elements using SEM-EDX. EDX helped to identify whether the three silver, zinc, and copper metal particles were successfully grafted onto the porous calcium oxide particles. In Figure 2c, we can find that the elements, calcium and oxygen, which is calcium oxide itself, are evenly distributed in the marked area. In contrast, the three silver-modified metals, zinc, and copper elements can also be observed in the significant area; in particular, the strength of the silver component of the area is marked very clearly. The above results support the successful grafting of the three silver, zinc, and copper metal particles onto calcium oxide powder.

### 3.2. Linear Low-Density Polyethylene (LLDPE) with Metal Ion (Me)-Containing CaO Film

#### 3.2.1. Physical and Chemical Structure Investigation

**Figure 3:**
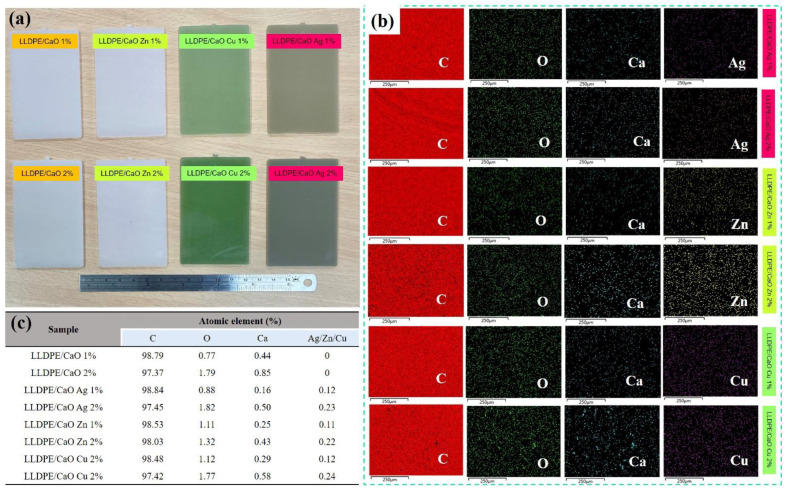
(**a**) Photograph of linear low-density polyethylene (LLDPE) added with different concentrations prepared by calcium oxide. (**b**) EDX spectra wide range of LLDPE/CaO- and LLDPE/CaO-containing metal ions (Ag, Zn, Cu). (**c**) atomic percentage data from EDX measurement.

After successfully obtaining CaO from OSP modified with metal ions (Me), the next step was to make a hybrid composite by mixing LLDPE (linear low-density polyethylene) and CaO from OSP changed with Me (Ag, Zn, and Cu). Figure 3 shows the hybrid composite sections of LLDPE mixed with different concentrations (1% and 2%) of Ag-, Zn-, and Cu-modified CaO. From the figure, it can be observed that the pieces of unmodified PE/CaO have a cream-colored appearance similar to the color of pure CaO powder. Likewise, LLDPE sheets with CaO-Me (Ag, Zn, and Cu) have the same color as the resulting powder. In addition, we observed that the increase in CaO/Me concentration change the color to being darker. In addition, the thickness for all the sheets (LLDPE/CaO-Ag, Zn, and Cu) was 0.2 cm. Basic LLDPE has a tensile strength of 130 kg/cm^2^ and an elongation break of 500%. The LLDPE strength test was carried out using the ASTM D638 method.

The subsequent analysis is the surface morphology test on LLDPE, mixed with CaCO_3_-metal and CaO-Me using SEM measurement. Figures S2 and S3 show that all surfaces of the mixed matrix film of CaCO_3_-Me and CaO-Me are flat and uniform. Further observation revealed small white spots on the film’s surface. The white areas were indicated by a mixture of oyster shell powder with modified metal ions. This modified Me-OSP is added to polyethylene. It migrates to the film’s surface during the process of screw granulation and hot pressing. In addition, the oyster shell powder was evenly distributed on the film’s surface, and no aggregation was observed. The modified metal ion–oyster shell powder was compatible with the polyethylene polymer interface.

**Figure 4:**
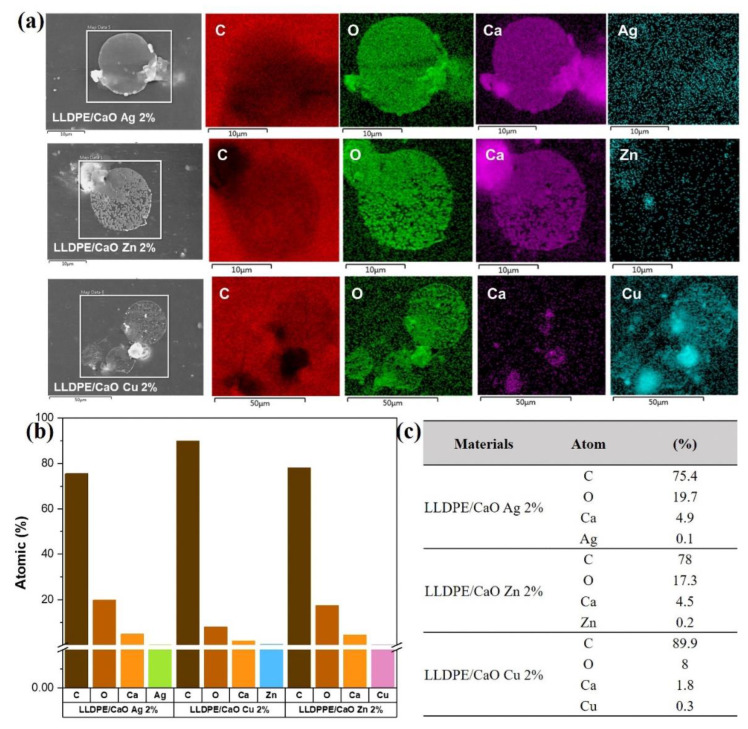
(**a**) Energy-dispersive X-ray spectroscopy (EDX) mapping of a small area of LLDPE/CaO Ag 2%, LLDPE/CaO Zn 2%, and LLDPE/CaO Cu 2%. (**b**) the atomic percentage of LLDPE/CaO Ag 2%, LLDPE/CaO Zn 2%, and LLDPE/CaO Cu 2%. (**c**) atomic percentage data from EDX measurement.

The subsequent measurement examined the chemical elements in the oyster shell-modified metal ion (Me) and LLDPE sheet using energy-dispersive X-ray spectroscopy (EDX). Figures S4 and S5 show the EDX spectra of LLDPE/CaCO_3_-containing Me (Ag, Zn, Cu) and LLDPE/CaO, respectively. It was found that the atomic elements of C, O, Ca, and Me were uniformly dispersed. As we can see from the quantitative data in Tables S2 and S3, because the oyster shell powder is added to the carbon-based polyethylene polymer body, the carbon elemental composition in the EDX elemental analysis is higher than 97%. Furthermore, it was found that with the addition of the three metal ions to the oyster shell powder, the content of each metal element Ag, Zn and Cu also increased, and the proportion of the metal element content increased in line with the modified metal ion–oyster shell powder. The amount of powder added to polyethylene was 1–2%, and the ratio of the various metal ion elements in the material increased from 0.1% to 0.2%. In addition, we analyzed a small particle of LLDPE/CaO-containing Me (Ag, Zn, Cu) with 2% concentration (see Figure 4); the EDX element was also uniformly dispersed and mixed in the polyethylene polymer, especially for metal ions such as Ag, Zn, and Cu.

**Figure 5:**
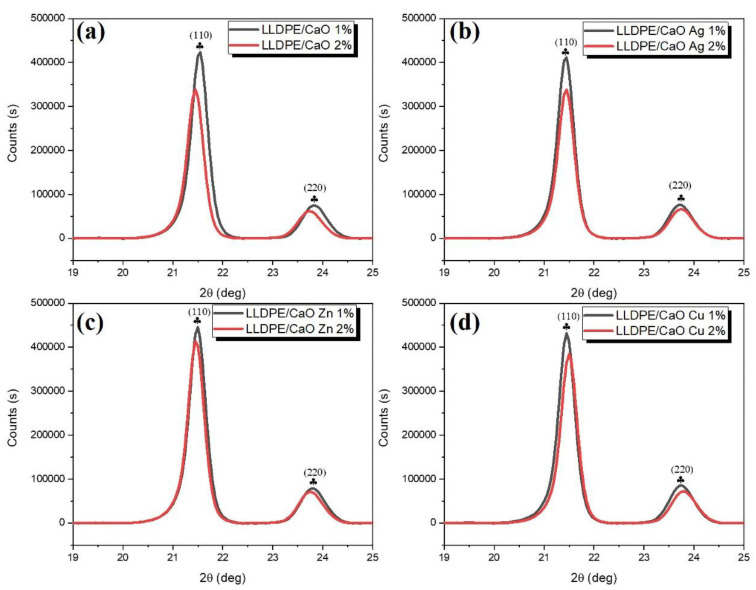
Wide-angle X-ray diffraction (WAXRD) pattern (19–25 deg) of (**a**) LLDPE/CaO, (**b**) LLDPE/CaO Ag, (**c**) LLDPE/CaO Zn, and (**d**) LLDPE/CaO Cu.

To confirm the degree of crystallinity (CD), we measured it using XRD diffractograms. Figure 5 and Figure S6 show XRD diffractograms of the LLDPE hybrid composite mixed with CaO-Me (Ag, Zn, Cu). Two distinct peaks at 21.5° and 23.7° correspond to the reflection planes of (110) and (220), respectively. The XRD pattern was convoluted to obtain more quantitative information with a Gaussian function whose full width at half maximum (FWHM) was determined from the Debye–Scherrer formula [47,48]. The size is inversely proportional to the full width at half maximum. To be more precise, the value of FWHM depends on the length over which the periodicity of the crystal is complete.

Based on the outcomes derived from WAXRD, the prominent characteristic peaks of LLDPE appeared to have been retained in all samples, indicating that the incorporation of coupled CaO/Ag, Zn, and Cu nanoparticles did not significantly alter the general structure of the LLDPE matrix. In contrast, the interaction between oxide nanoparticles and LLDPE involved a physical process. However, patterns of LLDPE show a remarkable degree of crystallinity. Planes (110) and (220) were used to determine the average degree of crystallinity and crystallite size of LLDPE nanocrystals. The average crystallite size was determined from the broadening of the corresponding peaks by using Scherrer’s. Table S4 shows the degree of crystallinity and crystallite size of the LLDPE composites increased with the addition of oyster shell powder to CaO and Me (Ag, Zn, Cu).

#### 3.2.2. Thermal Investigation

**Figure 6:**
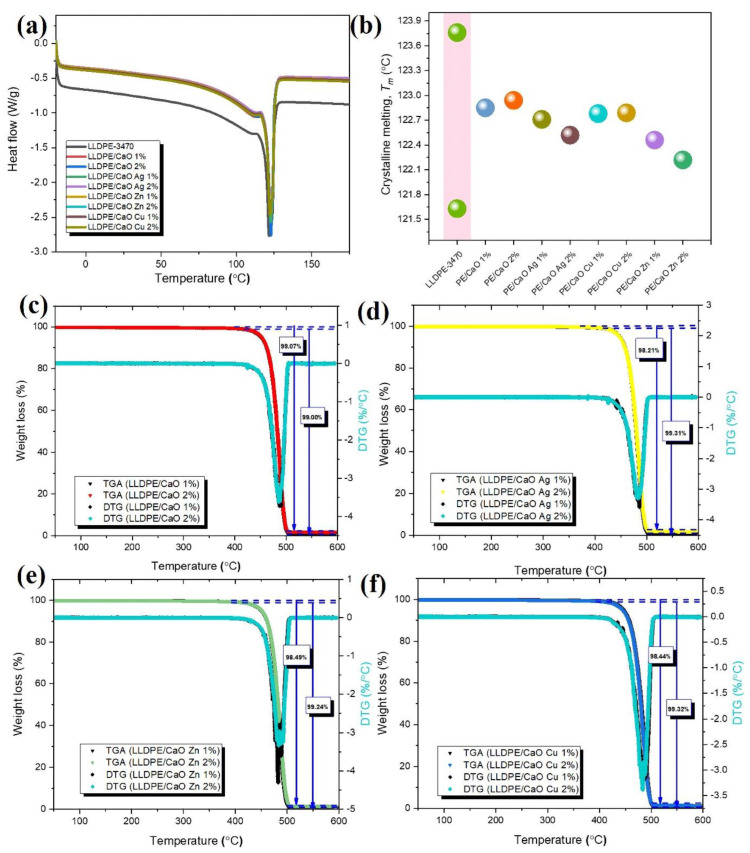
(**a**) DSC curves in the glass transition region of LLDPE/CaO, LLDPE/CaO Ag, LLDPE/CaO Zn, and LLDPE/CaO Cu against filler content (wt%), (**b**) T_m_ values. Thermogravimetric analysis (TGA, DTG) traces of (**c**) LLDPE/CaO, (**d**) LLDPE/CaO Ag, (**e**) LLDPE/CaO Zn, and (**f**) LLDPE/CaO Cu against filler content (wt%).

The DSC was introduced to investigate the composite’s crystalline melting (T_m_). Figure 6a,b shows the DSC thermograms of LLDPE with and without CaO–different metal ions and melting temperature (T_m_). From the T_m_ data, LLDPE has two melting points, at about 121.63 °C and 123.74 °C. However, for 1% and 2% of CaO, and CaO with the metal ions of the mixed LLDPE, each DSC trace comprises a single, broad peak with a melting temperature of around 122 °C. From DSC analysis, oyster shell powder containing the different ions, Ag, Zn, and Cu, did not strongly affect the melting point. Therefore, the hybrid composite LLDPE with OSP-Me has a good potential for industrial production and low energy consumption.

Ash testing is a common technique used to determine the composition of materials. By heating a sample to a high temperature in the air, all organic substances pyrolyze, leaving behind only inert and inorganic substances. This residue is weighed to determine its weight percentage in the original material. Thermogravimetric analysis (TGA) is commonly used for this purpose. The following application note discusses this technique and some typical examples of materials analyzed [49]. Here, it was used to investigate the thermal decomposition of LLDPE and LLDPE with CaO (Ag, Zn, Cu) blends with different percentages. The results of representative TGA and DTG tests of these materials are presented in Figure 6c–f.

The measured residue for LLDPE/CaO 1% at 600 °C was about 1% lower than 2% CaO (see Table S5). These results are probably within the process limits for this material, and the ash residue will be more when the CaO concentration is higher. Moreover, the stability of LLDPE did not appear to change significantly in the TGA curves of the metal ion biocomposites containing LLDPE/CaCO_3_ and LLDPE/CaO, as well as the decomposition temperatures of all specimens corresponding to the differential curves. Deviations were large enough to affect the mechanical properties. The thermal decomposition and mass loss for various mixtures of LLDPE mixed with CaO and Me were also investigated. Figure 6c–f and Figure S8 and Table S4 present the mass loss as a function of temperature for the test objects. The mass loss of LLDPE was 99.99%. However, we noticed that the LLDPE/CaO mixture, with 98% of the weight of LLDPE, underwent severe decomposition at 500 °C with a mass loss of more than 90%. In addition, the mixtures of LLDPE with CaO and Me (Ag, Zn, Cu) also showed the same mass loss at that temperature. All data confirm that CaO and metal ions mixed with LLDPE have good stability and their thermal stability is not affected.

**Figure 7:**
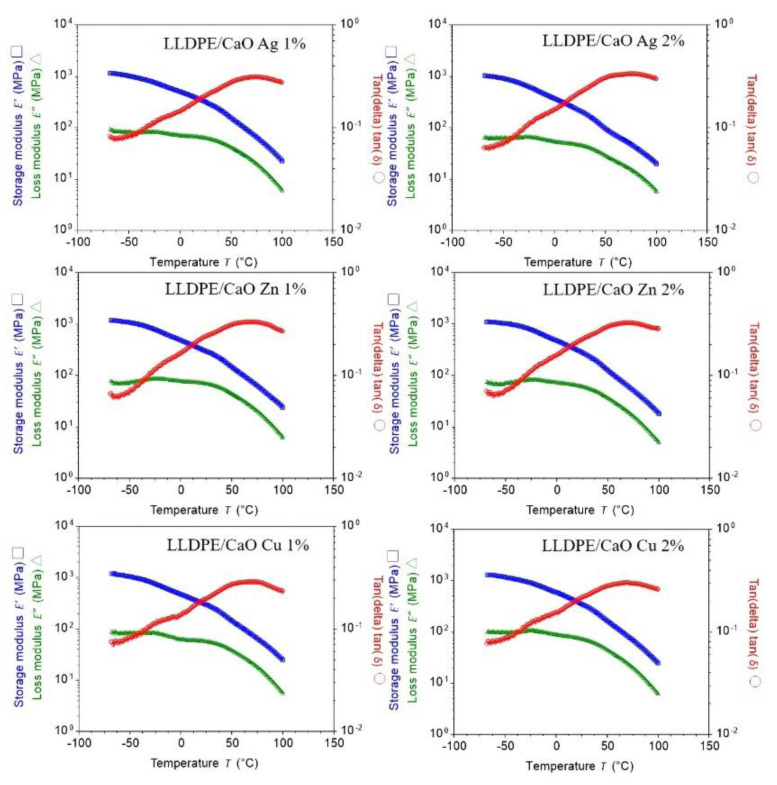
Typical DMA thermogram of PE/CaO with a different metal ion (Ag, Zn, Cu). Storage modulus (E′) and loss modulus (E″), and loss factor tan (delta) are plotted as a function of temperature.

The storage modulus (E′) and loss modulus (E′′) of adhesives were measured using DMA. Tan delta was calculated for these components, and its dependence on the temperature was plotted [50]. The temperature dependence of the storage modulus and the loss modulus of the sample formed are shown in Figure 7 and Figure S7. The figure is completed with a calculated tan delta. Curing in a vacuum improves the mechanical properties and decreases the mechanical losses. Fixing in a vacuum caused a decrease in the coefficient of thermal expansion and a reduction in the glass transition temperature as well. For all samples that have the same significance, we assume that adding oyster shell powder does not reduce the membrane strength at a given temperature.

#### 3.2.3. Antibacterial Investigation

**Figure 8:**
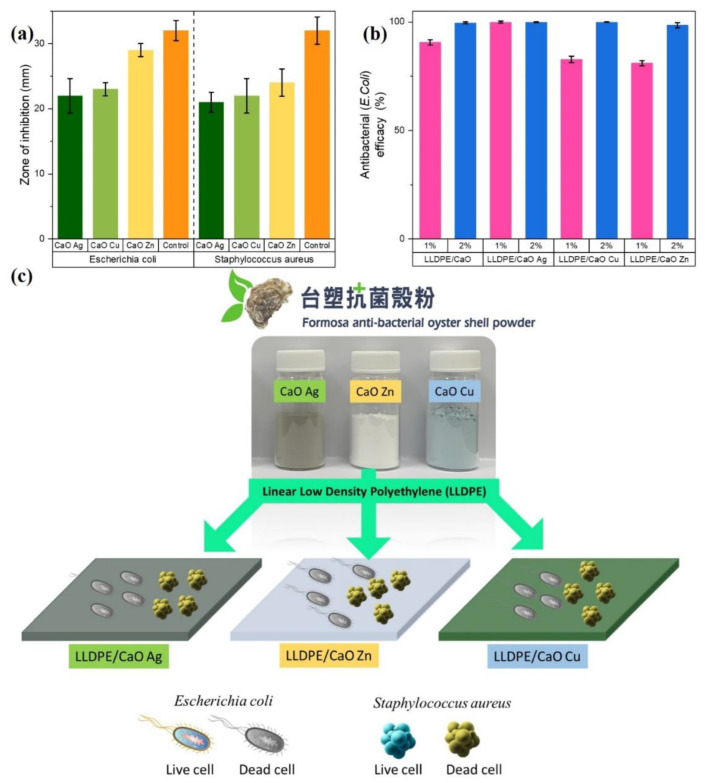
Antibacterial test of (**a**) modified metal ion–oyster shell powder and (**b**) mixing with LLDPE. (**c**) Schematic animation of antibacterial activity of *Escherichia coli* (*E. coli*) and *Staphylococcus aureus* (*S. aureus*).

Before antibacterial treatment on hybrid composite sheets, the antibacterial ring test of gram-positive *Staphylococcus aureus* (*S. aureus*) and gram-negative *Escherichia coli* (*E. coli*) was first tested on oyster shell powder. The results shown in Figure 8a and Table S6 and Table S7 show that the CaO powders grafted with metal ion particles are resistant to *E. coli* and *S. aureus*. The experimental results of the inhibition zone diameter of the bacteria show that the oyster shell powder had an excellent antibacterial effect, and the zone of inhibition of various modified metals was between 22–29 mm for *E. coli* and between 21–24 mm for *S. aureus*. Based on research that has been carried out, the broad categorization of inhibition zones consists of strong, middle, weak, and not active (Table S8). The 10–20 mm inhibition zone area is categorized as moderate, while a CaO and CaO-modified Ag, Zn, and Cu inhibition zone larger than 20 mm is categorized as strong. The above-mentioned modified Me-OSP showed an antibacterial effect on *E. coli* and *S. aureus*, especially on Zn metal. Therefore, we prepared pieces of the hybrid composites’ sheet to test its antibacterial efficacy by mixing the modified Me-OSP with LLDPE in different amounts (1% and 2%).

After testing the antibacterial inhibition on oyster shell powder, both modified and unmodified using metal ions. The inhibition zone was categorized as strong for all CaO-Me because the value was greater than 20 mm. Next, we tested the antibacterial efficacy of *E. coli* based on a modified LLDPE/metallic oyster shell biocomposite sheet. We used the Japanese industry-standard method (JIS Z 2801) to test the antibacterial efficacy against LLDPE/CaO surface sheets. (JIS) Z 2801 is a quantitative antimicrobial test that is commonly requested for plastics and is harmonized with ISO 22196 [9,51]. Various metal ions, Ag, Zn, and Cu–oyster shell powder, have an effective antibacterial ring for *E. coli*. This metal oyster–ion shell powder with an antibacterial effect was added and mixed in the polyethylene polymer, adding 1% and 2%. The antibacterial test pieces were prepared under the premise of not affecting the physical properties of the polymer material itself. From the test results for the antibacterial levels of *E. coli* on CaO without metal ions and LLDPE/CaO specimens, it can be seen that the addition of CaO-Me with LLDPE increases their antibacterial efficacy. It can be seen from Figure 8b and Table S7 that the antibacterial efficacy of pure CaO is lower than that of mixed Ag, Zn, and Cu. The antibacterial efficacy of LLDPE/CaO 1% is only 90.6%, but when it is mixed with Ag, the efficacy increases to 99.9%. Adding oyster shell and metal ion powder, of as much as 2%, in LLDPE has a more significant effect, as evidenced by the overall efficacy increase to more than 99.9%. Moreover, adding metal ions can strengthen antibacterial efficacy, especially Ag metal ions. Even though the concentration is small, the antibacterial efficacy is still 99.9%. This is because silver ions (Ag) are very good at resisting the growth of gram-negative *E. coli* [52,53]. These results show that the Me-modified OSP significantly affects the antibacterial LLDPE sheet (see Figure 8c).

The results of these studies provide evidence that CaO from OSP has the potential to be used as a powerful antimicrobial agent for manufacturing safe food and medicinal products. Radheshkumar and Munstedt [53] studied and explained the release process for Ag ions from a polymer matrix. This behavior is composed of three elementary processes: water diffusion in the composite specimen; the reaction between the nanoparticles and water molecules, leading to the formation of Ag ions; and the migration of Ag ions through the composite specimen, leading to their release from the composite to the aqueous environment. The release of silver ions requires their diffusion through the corresponding amorphous portions of the polymer. The release analysis is related to the antimicrobial properties.

## 4. Conclusions

In conclusion, this study modified LLDPE by mixing it with oyster shell powder CaO from oyster shell powder (OSP) and different metal ions (Ag, Zn, and Cu). All ingredients were mixed evenly, as indicated by the SEM-EDX measurement results. All surfaces of the LLDPE/CaO-Me hybrid composite looked smooth. With the EDX test, it was seen that the percentage of metal ions and CaO increased with increasing concentration (1% and 2%). Atomic elements for metal ions increased by 0.23%, 0.22%, and 0.24% for Ag, Zn, and Cu, respectively. On antibacterial assays, the zones of inhibition of various modified metals were 22–29 mm for *E. coli* and between 21 and 24 mm for *S. aureus*. Linear low-density polyethylene (LLDPE) combined with CaO and metal ion forms can be an excellent alternative to solid hybrid composites. The strength modulus at 1% LLDPE to LLDPE/CaO Ag increased from 297 MPa to 320 MPa. The degree of crystallinity of LLDPE also increased from 35% to 37.7%. In addition, these sheets (LLDPE/CaO Ag, LLDPE/CaO Zn, and LLDPE/CaO Cu) were excellent and functioned as antibacterial agents against *E. coli* with an antibacterial rate of 99.9%.

## Data Availability

All data generated or analyzed during this study are included in this published article (and its supplementary information files).

## Supplementary Materials

The following supporting information can be downloaded at: https://www.mdpi.com/article/10.3390/polym14153001/s1, Figure S1: Datasheet of oyster shell powder (OSP) masterbatch by Formosa plastics corporation; Figure S2: SEM surface image of polypropylene test pieces added with different concentrations prepared by calcium carbonate series modified metal ions; Figure S3: SEM surface image of polypropylene test pieces added with different concentrations prepared by calcium oxide series modified metal ions; Figure S4: EDX spectra of LLDPE/CaCO_3_ and LLDPE/CaCO_3_ containing metal ions (Ag, Zn, Cu); Figure S5: EDX spectra of LLDPE-3470 and LLDPE/CaO with different concentrations (1% and 2%); Figure S6: XRD pattern of samples and degree of crystallinity (CD); Figure S7: Typical DMA thermogram of LLDPE with different (%) of CaO. Storage Modulus (E′) and Loss Modulus (E″), and Loss Factor tan(delta) are plotted as a function of temperature; Figure S8: Thermogravimetric analysis (TGA) of LLDPE-3470; Table S1: Composition of LLDPE-3470 biocomposites; Table S2: SEM-EDX mapping elemental analysis of LLDPE added with different concentrations of CaCO_3_ series modified metal ions; Table S3: The atomic percentage of LLDPE/CaO data from EDX measurement; Table S4: Position of crystalline peaks and crystal indices for LLDPE and LLDPE nanocomposites with different ratios of CaO/Ag, Zn, Cu nanoparticles; Table S5: Thermogravimetric analysis (TGA) analysis data of LLDPE and LLDPE biocomposites with different ratios of CaO/Ag, Zn, Cu nanoparticles; Table S6: antibacterial activity (*Escherichia coli* and *Staphylococcus aureus*) of powder CaO-Metal ions; Table S7: Antibacterial efficacy (%) *E. coli* of LLDPE/CaO 1% and LLDPE/CaO 2% with a deferent metal ion; Table S8 [54,55]: Classification antimicrobial sensitivity.
