# Supplementary material for: Evaluation of the Antibacterial Activity of Eco-Friendly Hybrid Composites on the Base of Oyster Shell Powder Modified by Metal Ions and LLDPE
Source: Polymers (Basel). 2022 Jul 25;14(15):3001. doi: 10.3390/polym14153001 (PMC9332488; doi:10.3390/polym14153001)
Supplement: Supplementary file 1 [file polymers-14-03001-s001.zip › polymers-1807141-supplementary.pdf]

SUPPORTING INFORMATION

Evaluation of the antibacterial activity of eco-friendly hybrid composites on the base of oyster shell powder modified by metal ions and LLDPE.

Januar Widakdo [1], Tsan-Ming Chen [2], Meng-Chieh Lin [2], Jia-Hao Wu [2], Tse-Ling Lin [2], Pin-Ju Yu [2], Wei-Song Hung [1,3]* Kueir-Rarn Lee [3]*

[1]Advanced Membrane Materials Research Center, Graduate Institute of Applied Science and Technology, National Taiwan University of Science and Technology, Taipei, 106335, Taiwan.

[2] Carbide Division, Formosa Plastics Corporation, Taipei, 105076, Taiwan

[3] R&D Center for Membrane Technology, Department of Chemical Engineering, Chung Yuan University, Chung Li, 32023, Taiwan

* Email: *wshung@mail.ntust.edu.tw* and *krlee@cycu.edu.tw*

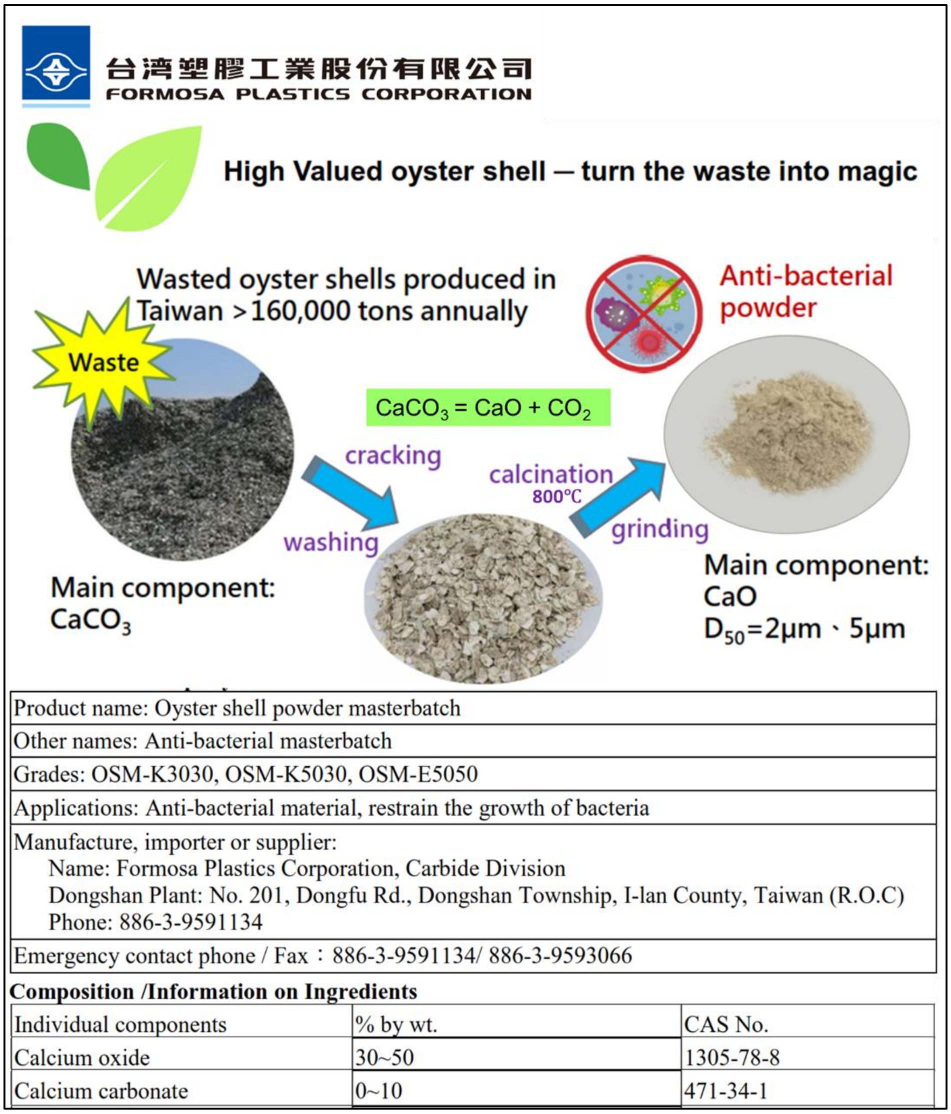

Product name: Oyster shell powder masterbatch	
Other names: Anti-bacterial masterbatch	
Grades: OSM-K3030, OSM-K5030, OSM-E5050	
Applications: Anti-bacterial material, restrain the growth of bacteria	
Manufacture, importer or supplier:<br>Name: Formosa Plastics Corporation, Carbide Division<br>Dongshan Plant: No. 201, Dongfu Rd., Dongshan Township, I-lan County, Taiwan (R.O.C)<br>Phone: 886-3-9591134	
Emergency contact phone / Fax：886-3-9591134/ 886-3-9593066	

Composition /Information on Ingredients

Individual components	% by wt.	CAS No.
Calcium oxide	30~50	1305-78-8
Calcium carbonate	0~10	471-34-1

Figure S1 Datasheet of oyster shell powder (OSP) masterbatch by Formosa plastics corporation

Table S1. Composition of LLDPE-3470 biocomposites.

Sample name	Composition				
	LLDPE (gram)	CaO (gram)	CaO-Ag (gram)	CaO-Zn (gram)	CaO-Cu (gram)
LLDPE-3470	100	-	-	-	-
LLDPE/CaO 1%	99	1	-	-	-
LLDPE/CaO 2%	98	2	-	-	-
LLDPE/CaO Ag 1%	99	-	1	-	-
LLDPE/CaO Ag 2%	98		2	-	-
LLDPE/CaO Zn 1%	99	-	-	1	-
LLDPE/CaO Zn 2%	98	-	-	2	-
LLDPE/CaO Cu 1%	99	-	-	-	1
LLDPE/CaO Cu 2%	98	-	-	-	2

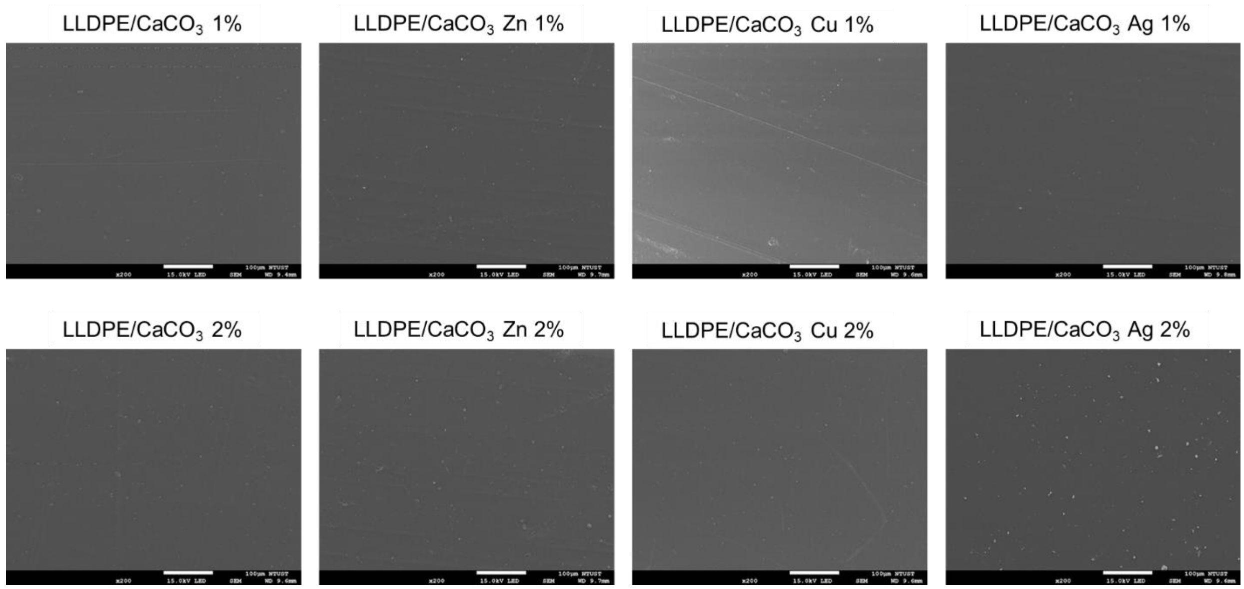

Figure S2, SEM surface image of polypropylene test pieces added with different concentrations prepared by calcium carbonate series modified metal ions

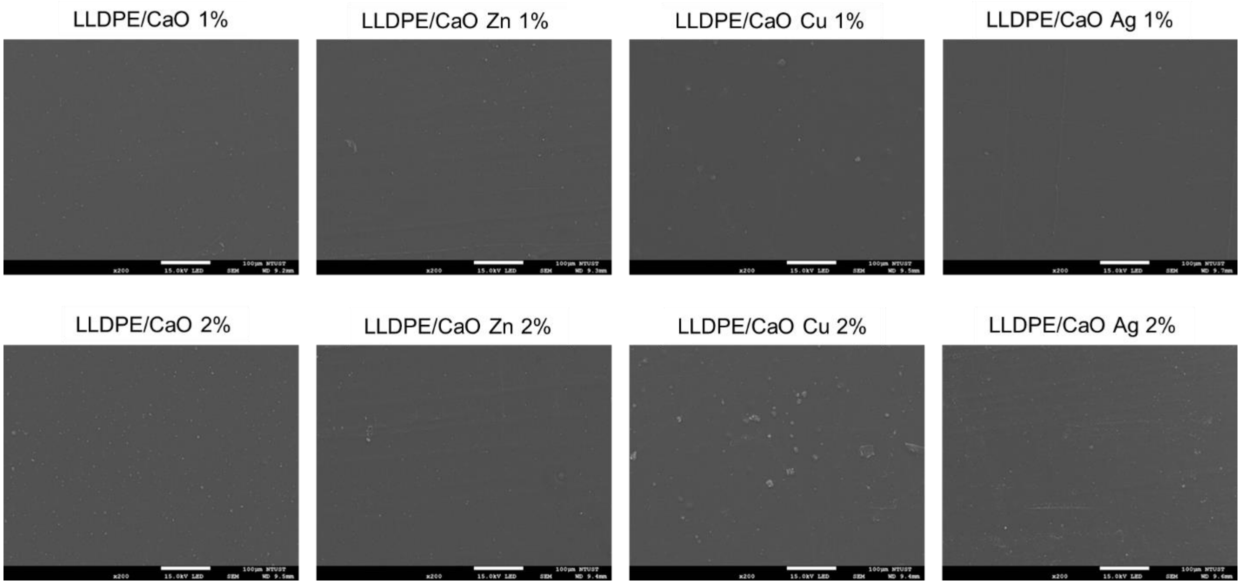

Figure S3, SEM surface image of polypropylene test pieces added with different concentrations prepared by calcium oxide series modified metal ions.

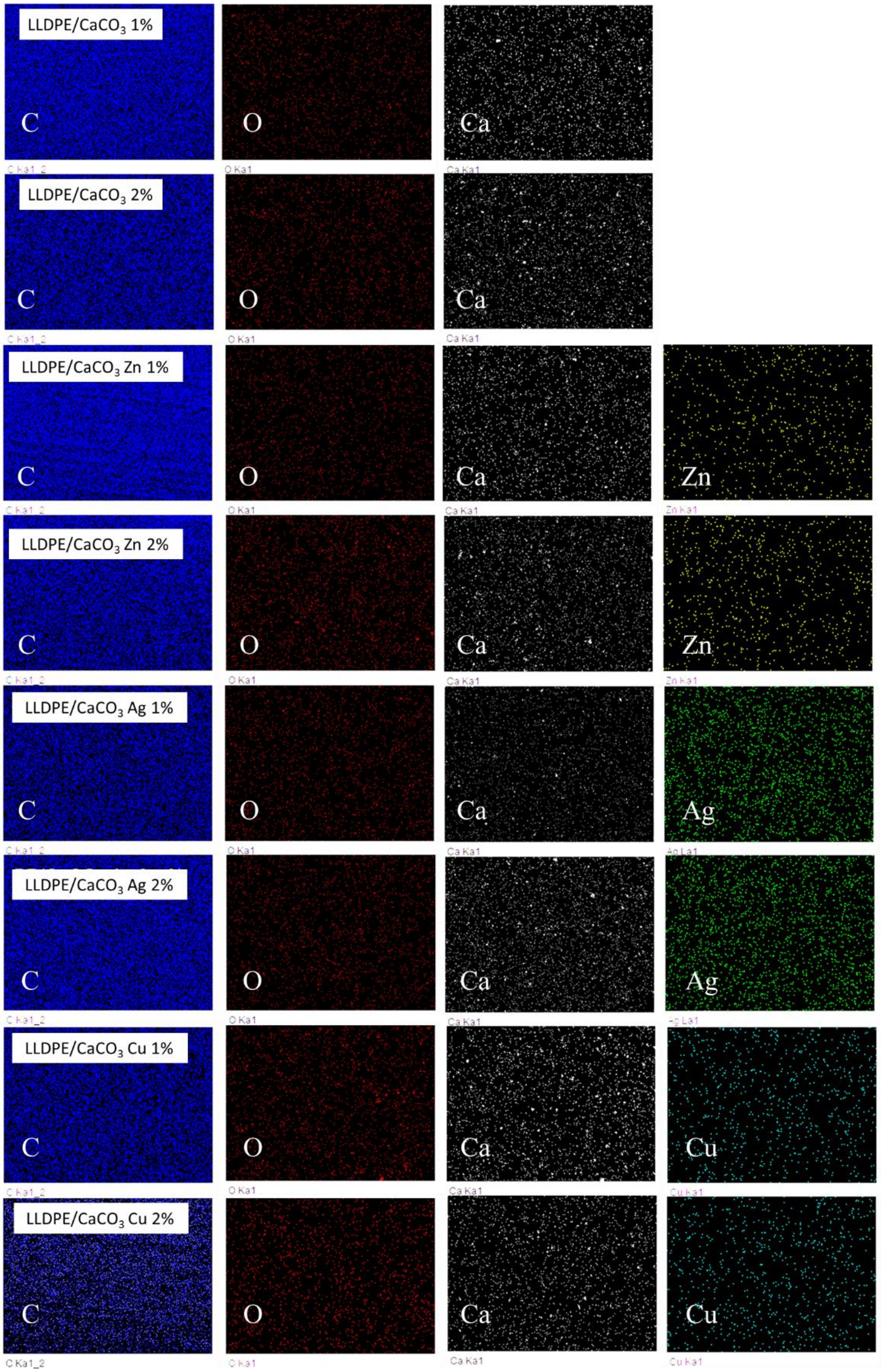

Figure S4 EDX spectra of LLDPE/$CaCO_3$ and LLDPE/ $CaCO_3$ containing metal ions (Ag, Zn, Cu).

Table S2. SEM-EDX mapping elemental analysis of LLDPE added with different concentrations of $CaCO_3$ series modified metal ions

Sample	Atom (%)				Total
	C	O	Ca	Zn/Ag/Cu	
LLDPE/$CaCO_3$ 1%	98.14	1.30	0.57	0	100 %
LLDPE/$CaCO_3$ 2%	97.49	2.09	0.42	0	100 %
LLDPE/$CaCO_3$ Zn 1%	98.57	1.18	0.16	0.10	100 %
LLDPE/$CaCO_3$ Zn 2%	97.95	1.47	0.36	0.23	100 %
LLDPE/$CaCO_3$ Cu 1%	98.38	1.21	0.29	0.11	100 %
LLDPE/$CaCO_3$ Cu 2%	97.83	1.46	0.52	0.25	100 %
LLDPE/$CaCO_3$ Ag 1%	98.38	1.41	0.14	0.12	100 %
LLDPE/$CaCO_3$ Ag 2%	97.86	1.59	0.36	0.24	100 %

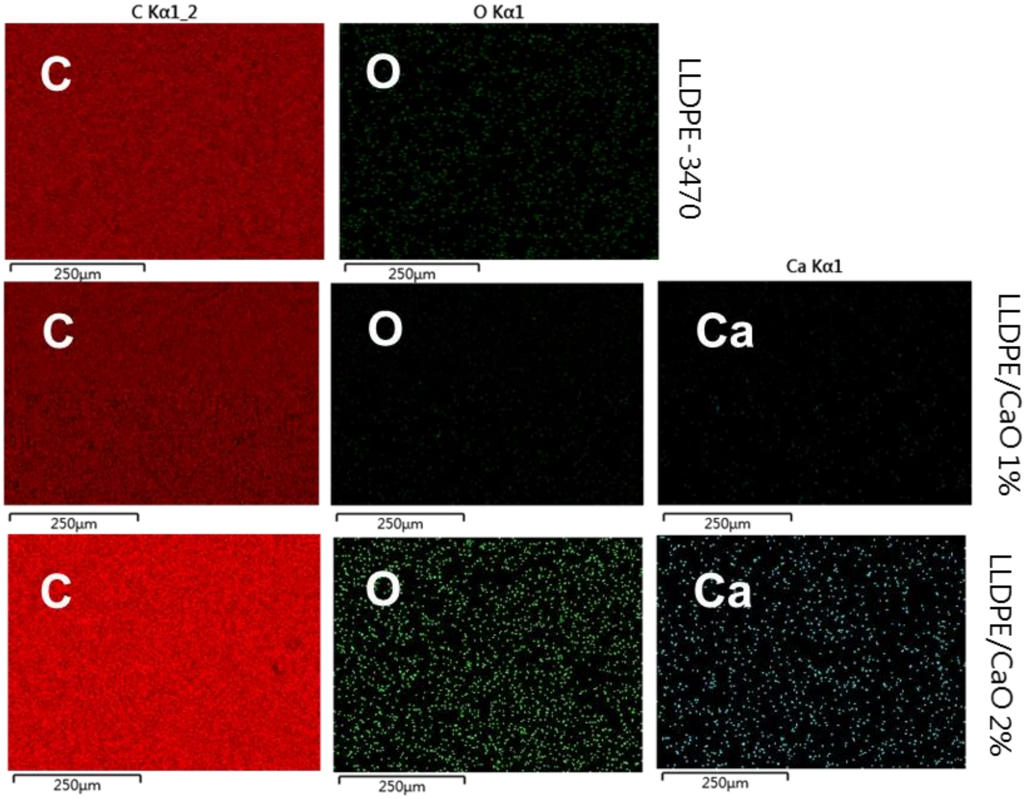

Figure S5 EDX spectra of LLDPE-3470 and LLDPE/CaO with different concentrations (1% and 2%).

Table S3. The atomic percentage of LLDPE/CaO data from EDX measurement

Sample	Atom (%)		
	C	O	Ca
LLDPE-3470	99.4	0.6	0
LLDPE/CaO 1%	98.4	1.1	0.5
LLDPE/CaO 2%	97.6	1.6	0.8

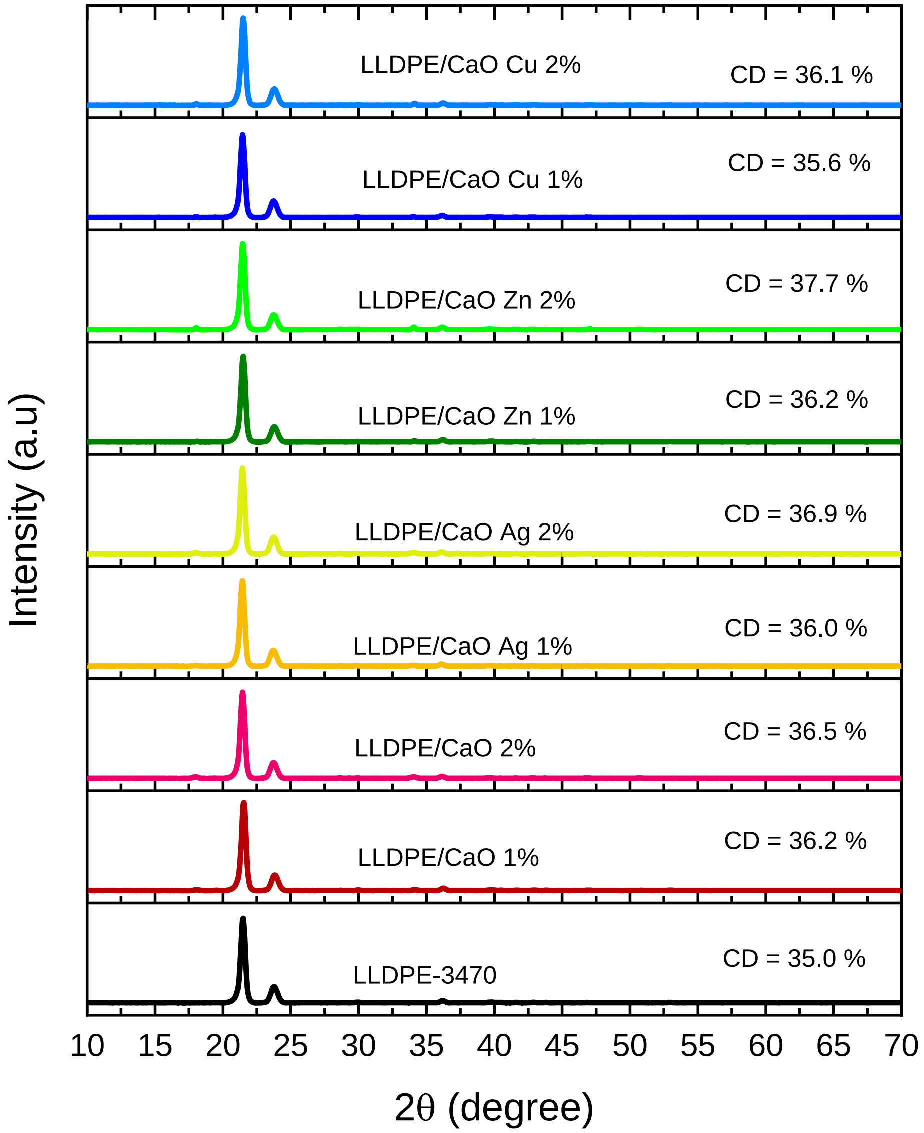

Figure S6 XRD pattern of samples and degree of crystallinity (CD)

Table S4. Position of crystalline peaks and crystal indices for LLDPE and LLDPE nanocomposites with different ratios of CaO/Ag, Zn, Cu nanoparticles

Sample	Peak 2θ (°)		FWHM β		Crystallite Size (nm)		d-spacing (nm)		Degree of Crystallinity X (%)
	110	220	110	220	110	220	110	220	
LLDPE	21.48	23.78	0.42	0.58	20.00	14.66	4.13	3.74	35
LLDPE/CaO 1%	21.53	23.83	0.42	0.58	19.93	14.71	4.12	3.73	36.2
LLDPE/CaO 2%	21.45	23.74	0.42	0.57	20.26	14.96	4.14	3.74	36.5
LLDPE/CaO Ag 1%	21.43	23.73	0.42	0.57	20.21	14.84	4.14	3.75	36
LLDPE/CaO Ag 2%	21.44	23.75	0.42	0.57	20.12	14.90	4.14	3.74	36.9
LLDPE/CaO Zn 1%	21.49	23.80	0.42	0.57	19.99	14.78	4.13	3.74	35.6
LLDPE/CaO Zn 2%	21.46	23.76	0.42	0.57	20.15	14.89	4.14	3.74	36.1
LLDPE/CaO Cu 1%	21.45	23.75	0.42	0.58	20.15	14.64	4.14	3.74	36.2
LLDPE/CaO Cu 2%	21.49	23.80	0.42	0.42	20.24	20.32	4.13	3.74	37.7

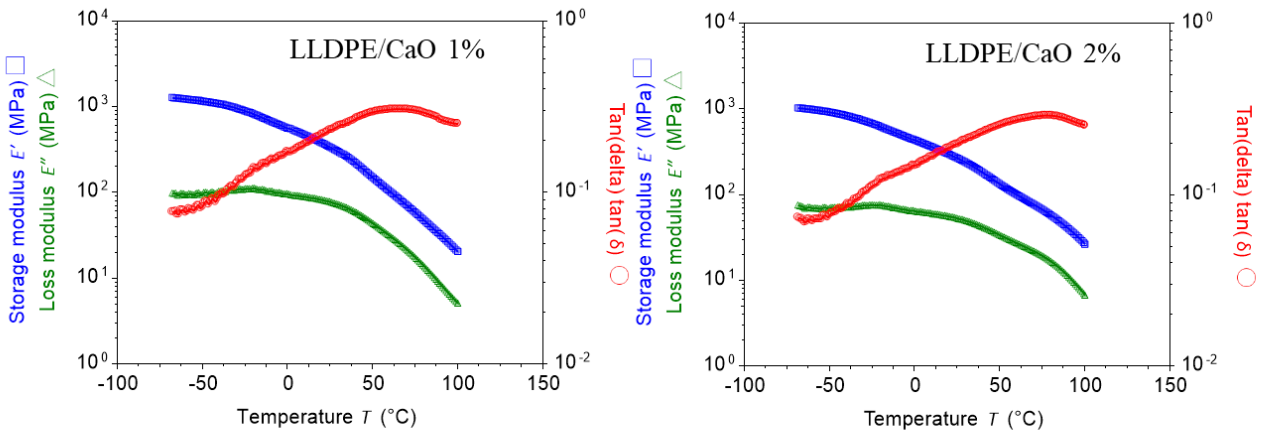

Figure S7. Typical DMA thermogram of LLDPE with different (%) of CaO. Storage Modulus (E') and Loss Modulus (E''), and Loss Factor tan(delta) are plotted as a function of temperature.

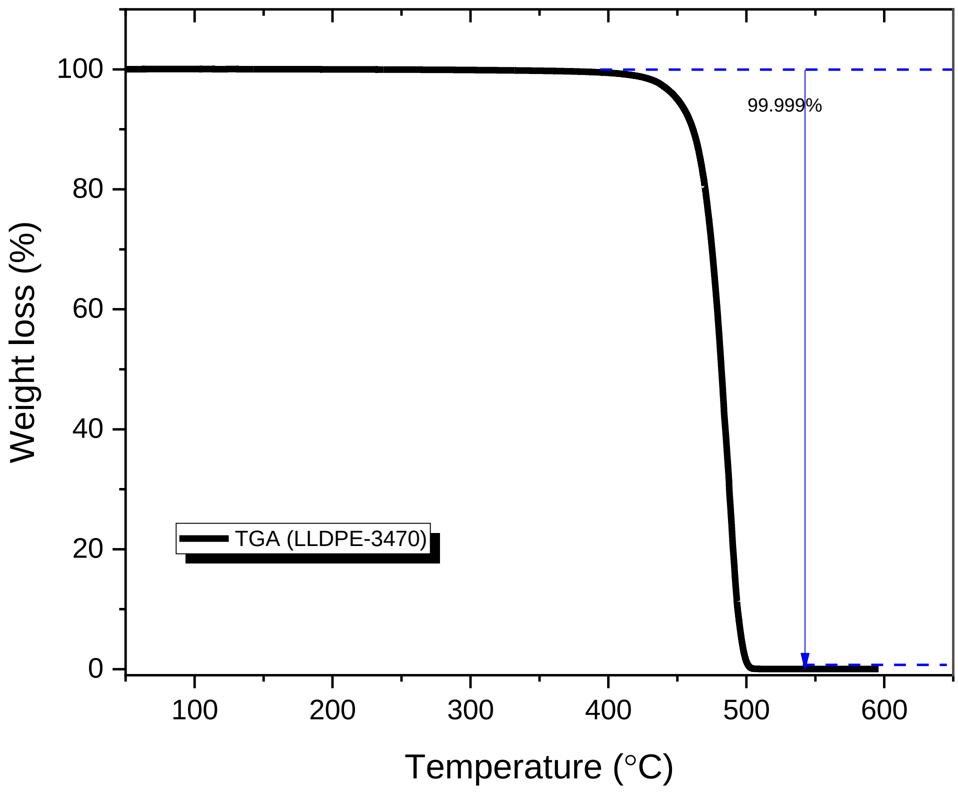

Figure S8. Thermogravimetric analysis (TGA) of LLDPE-3470.

Table S5 Thermogravimetric analysis (TGA) analysis data of LLDPE and LLDPE biocomposites with different ratios of CaO/Ag, Zn, Cu nanoparticles

	Sample	Ash testing residue (%)	Mass loss (%)
**Thermogravimetric analysis (TGA)**	LLDPE-3470	0.00	99.999
	LLDPE/CaO 1%	0.85	99.008
	LLDPE/CaO 2%	1.78	98.077
	LLDPE/CaO Ag 1%	0.66	99.307
	LLDPE/CaO Ag 2%	1.39	98.211
	LLDPE/CaO Zn 1%	0.72	99.214
	LLDPE/CaO Zn 2%	1.83	98.487
	LLDPE/CaO Cu 1%	0.53	99.320
	LLDPE/CaO Cu 2%	1.31	98.440

Table S6 antibacterial activity (*Escherichia coli* and *Staphylococcus aureus*) of powder CaO-Metal ions

Materials	Antibacterial ring, Inhibition zone diameter (mm)	
	Escherichia coli	***Staphylococcus aureus***
Control	32.00 ± 1.53	32.00 ± 2.08
CaO-Ag	22.00 ± 2.65	21.00 ± 1.53
CaO-Zn	29.00 ± 1.00	24.00 ± 2.08
CaO-Cu	23.00 ± 1.00	22.00 ± 2.65

Table S7 Antibacterial efficacy (%) *E.Coli* of LLDPE/CaO 1% and LLDPE/CaO 2% with a deferent metal ion.

	**Materials**							
	LLDPE/CaO		LLDPE/CaO Ag		LLDPE/CaO Zn		LLDPE/CaO Cu	
	1%	2%	1%	2%	1%	2%	1%	2%
**Antibacterial efficacy (%)** ***E.Coli***	90.60 ± 1.15	99.5 ± 0.46	99.9 ± 0.49	99.9 ± 0.15	81 ± 1.15	98.5 ± 1.15	82.7 ± 1.53	99.9 ± 0.10

Table S8. Classification antimicrobial sensitivity[1, 2]

Diameter Zone of inhibition (mm)	Antimicrobial activity
≤ 10	Not active
11-15	Weak
16-20	Middle
≥ 20	Strong

References

1. Indriani, V., et al., *Antibacterial Effect of Curcuma zedoaria Extract on Bacillus cereus and Staphylococcus epidermidis.* Althea Medical Journal, 2020. **7**(1): p. 6-10.
2. Zainuddin, A. and A.H. Saputro. *Antimicrobial effectiveness measurement system based on circle detection algorithm*. in *2017 International Seminar on Sensors, Instrumentation, Measurement and Metrology (ISSIMM)*. 2017. IEEE.

Parametric and Non-parametric tests

Analysis of variance (ANOVA) of LLDPE/CaO-Metal ion biocomposites.

Anova: Single Factor

SUMMARY

Groups	*Count*	*Sum*	*Average*	*Variance*
LLDPE/CaO 1%	3	270.3	90.1	1.33
LLDPE/CaO 2%	3	298.5	99.5	0.21
LLDPE/CaO Ag 1%	3	299.7	99.9	0.01
LLDPE/CaO Ag 2%	3	299.7	99.9	0.01
LLDPE/CaO Zn 1%	3	243	81	0.25
LLDPE/CaO Zn 2%	3	295.5	98.5	0.25
LLDPE/CaO Cu 1%	3	248.2	82.73333	2.413333
LLDPE/CaO Cu 2%	3	299.7	99.9	0.01

ANOVA

Source of Variation	*SS*	*df*	*MS*	*F*	*P-value*	*F crit*
Between Groups	1398.152	7	199.736	356.4062	2.38E-16	2.657197
Within Groups	8.966667	16	0.560417			
Total	1407.118	23				

Nonparametric Kruskal-Wallis Test of LLDPE/CaO-Metal ion biocomposites.

LLDPE/CaO 1%	LLDPE/CaO 2%	LLDPE/CaO Ag 1%	LLDPE/CaO Ag 2%	LLDPE/CaO Zn 1%	LLDPE/CaO Zn 2%	LLDPE/CaO Cu 1%	LLDPE/CaO Cu 2%
89	99	99.8	99.9	81	99	84	99.8
91.3	99.9	99.9	99.8	80.5	98	81	99.9
90	99.6	100	100	81.5	98.5	83.2	100

$$H = \left[\frac{12}{n_T(n_T+1)} \sum_{i=1}^{k} \frac{R_i^2}{n_i} \right] - 3(n_T + 1)$$

k = number of populations

n_i = the number of observations in sample i

$n_T = \sum_{i=1}^{k} n_i$ = the total number of observations in all samples

R_i = the sum of the ranks for sample i

**H**	20.46
**chi-square**	5.99
**p-value**	3.60E-05

Nonparametric Methods, Kruskal-Wallis Test

Analysis of variance (ANOVA) of CaO-Metal ion powder for *E.Coli* bacterial.

Anova: Single Factor

SUMMARY

Groups	Count	Sum	Average	Variance
CaO Ag (E.Coli)	3	66	22	7
CaO Zn (E.Coli)	3	87	29	1
CaO Cu (E.Coli)	3	69	23	4

ANOVA

Source of Variation	SS	df	MS	F	P-value	F crit
Between Groups	86	2	43	10.75	0.010386	5.143253
Within Groups	24	6	4			
Total	110	8				

Nonparametric Kruskal-Wallis Test of CaO-Metal ion powder for *E.Coli* bacterial.

CaO Ag (E.Coli)	CaO Zn (E.Coli)	CaO Cu (E.Coli)
20	29	21
21	30	23
25	28	25

$$H = \left[\frac{12}{n_T(n_T+1)}\sum_{i=1}^{k}\frac{R_i^2}{n_i}\right] - 3(n_T+1)$$

k = number of populations

n_i = the number of observations in sample i

$n_T = \sum_{i=1}^{k} n_i$ = the total number of observations in all samples

R_i = the sum of the ranks for sample i

**H**	5.60
**chi-square**	5.99
**p-value**	0.06

Analysis of variance (ANOVA) of CaO-Metal ion powder for *S. Aureus* bacterial.

Anova: Single Factor

SUMMARY

Groups	*Count*	*Sum*	*Average*	*Variance*
CaO Ag (S. Aureus)	3	64	21.33333	2.333333
CaO Zn (S. Aureus)	3	73	24.33333	2.333333
CaO Cu (S. Aureus)	3	66	22	1

ANOVA

Source of Variation	*SS*	*df*	*MS*	*F*	*P-value*	*F crit*
Between Groups	14.88889	2	7.444444	3.941176	0.080735	5.143253
Within Groups	11.33333	6	1.888889			
Total	26.22222	8				

Nonparametric Kruskal-Wallis Test of CaO-Metal ion powder for *S. Aureus* bacterial.

CaO Ag (S.Aureus)	CaO Zn (S. Aureus)	CaO Cu (S. Aureus)
20	26	21
23	23	23

$$H = \left[\frac{12}{n_T(n_T+1)} \sum_{i=1}^{k} \frac{R_i^2}{n_i} \right] - 3(n_T+1)$$

k = number of populations

n_i = the number of observations in sample i

$n_T = \sum_{i=1}^{k} n_i$ = the total number of observations in all samples

R_i = the sum of the ranks for sample i

**H**	4.47
**chi-square**	5.99
**p-value**	0.11
